# Does Artesunate Prolong the Electrocardiograph QT Interval in Patients with Severe Malaria?

**DOI:** 10.4269/ajtmh.2009.08-0326

**Published:** 2009-01

**Authors:** Richard J. Maude, Katherine Plewes, M. Abul Faiz, Josh Hanson, Prakaykaew Charunwatthana, Sue J. Lee, Joel Tärning, Emran Bin Yunus, M. Gofranul Hoque, Mahatab Uddin Hasan, Amir Hossain, Niklas Lindegardh, Nicholas P. J. Day, Nicholas J. White, Arjen M. Dondorp

**Affiliations:** Centre for Clinical Vaccinology and Tropical Medicine, Nuffield Department of Clinical Medicine, University of Oxford, Oxford, United Kingdom; Faculty of Tropical Medicine, Mahidol University, Bangkok, Thailand; Dhaka Medical College, Dhaka, Bangladesh; Chittagong Medical College Hospital, Chittagong, Bangladesh

## Abstract

Several antimalarials can cause significant prolongation of the electrocardiograph QT interval, which can be associated with an increased risk of potentially lethal ventricular arrhythmias. High doses of artemether and artemotil have been associated with QT prolongation in dogs, raising the possibility of a class effect with the artemisinin derivatives. Serial electrocardiograms were recorded, and QTc interval was calculated before and after administration of artesunate by intravenous injection in patients with severe falciparum malaria in Bangladesh. Of 21 adult patients with severe malaria enrolled, 8 (38%) died. The mean QTc interval was unaffected by bolus intravenous artesunate (2.4 mg/kg). In two patients, the QTc interval exceeded 0.5 seconds, but in both cases, an alternative explanation was plausible. No effect was observed on the JTc or PR interval, QRS width, blood pressure, or heart rate. Intravenous artesunate does not have significant cardiovascular effects in patients with severe *falciparum* malaria.

## Introduction

Artemisinin-based combination therapies (ACTs) are currently recommended worldwide as the first-line treatment for patients with uncomplicated *falciparum* malaria by the World Health Organization (WHO).^1^ Parenteral artesunate is the treatment of choice for adult patients with severe *falciparum* malaria.^1^,^2^ This extensive and often unsupervised deployment requires an excellent safety profile for this class of drugs. Several antimalarial drugs, notably quinidine and halofantrine, produce clinically significant delays in ventricular repolarization, resulting in a prolongation of the electrocardiographic QT interval on the electrocardiogram (ECG).^3^ Heterogeneous prolongation of ventricular repolarization predisposes to potentially lethal polymorphic malignant ventricular tachyarrhythmias (torsades de pointes [TdP]). The belated discovery that the antimalarial halofantrine causes marked QT prolongation and sudden death,^4^ well after its registration by several regulatory authorities, has focused attention on the potential cardiotoxicity of the antimalarial drugs. Regulatory authorities and drug developers have become particularly concerned about QT prolongation.^5^–^8^ The artemisinins are potent antimalarial drugs that are remarkably well tolerated. High intramuscular doses of the oil-based artemether and artemotil were associated with significant QT prolongation in toxicologic studies conducted in beagle dogs, raising the possibility of cardiotoxicity with this drug class.^9^ Information on the electrocardiographic effects of the artemisinin derivatives in humans is limited. In clinical studies reporting modest QT interval prolongation with the artemisinins, the contributions of drug and disease cannot be disentangled, because recovery from malaria itself is associated with significant lengthening of the QT interval.^3^,^10^ Volunteer studies do not suggest a significant cardiovascular effect. We therefore conducted a study on the acute effects of high-dose intravenous artesunate on the electrocardiographic intervals in patients with severe malaria, both on admission and after recovery.

## Materials and Methods

### Study site and patients.

This study was conducted at Chittagong Medical College Hospital (CMCH), a large 1,000-bed teaching hospital in Chittagong, Bangladesh. Malaria transmission is seasonal and of low intensity in this location. The study was part of our studies in severe malaria for which ethical approval was obtained from the Medical Research Council of Bangladesh.

Adult patients (≥16 years old) who had been admitted to hospital with a slide-confirmed diagnosis of severe malaria according to modified WHO criteria^10^ were recruited if they, or an attending relative, provided written informed consent. Criteria on admission for severe malaria included one or more of the following: cerebral malaria (Glasgow Coma Scale [GCS] <11), severe anemia (hematocrit <20% with parasite count >100,000/μL), jaundice (bilirubin >3.0 mg/dL with parasite count >100,000/μL), renal failure (serum creatinine >3 mg/dL), hypoglycemia (blood glucose <40 mg/dL), shock (systolic blood pressure <80 mm of Hg with cool extremities), hyperparasitemia (peripheral asexual stage parasitemia >10%), hyperlactatemia (venous plasma lactate >4 mmol/L), and acidemia (venous plasma bicarbonate <15 mmol/L). Pregnant or breast-feeding women and any patient who had received any cardioactive drugs within 1 week before the start of artesunate were excluded from the study.

### Drug treatment.

Antimalarial drug treatment was with intravenous artesunate (2.4 mg/kg body weight on admission, followed by 2.4 mg/kg at 12 and 24 hours and then every 24 hours; Guilin Pharmaceutical Factory, Guangxi, China). When the patient was able to take food, treatment was switched to oral artemether plus lumefantrine for a further 3 days.

Supportive treatments were in accordance with the 2000 guidelines of the WHO^1^ and local hospital guidelines, but the availability of renal replacement therapy and mechanical ventilation was limited.

### Study procedures.

On admission, a full history and examination were carried out. Blood samples were obtained for hemoglobin, hematocrit, parasitemia, platelet count, white cell count, plasma lactate levels, glucose levels, and full biochemistry. Patients were followed up a minimum of once daily until discharge, including a full and detailed neurologic examination where clinically indicated.

Serial 12 lead electrocardiograms were performed (electrocardiograph model SE-1; Edan Instruments, Shenzhen, China), with a paper speed of 50 mm/s and sensitivity of 20 mm/mV, immediately followed by a rhythm strip with 10 QRS complexes in each of leads II and V2. Time points for recording ECGs were immediately before artesunate administration (time 0) and at 10, 30, 60, 120, and 240 minutes after administration. At each time point, blood pressure, heart rate, and aural temperature were measured. Blood was collected on ice into fluoride/oxalate tubes at the same time points for assay of artesunate and dihydroartemisinin (DHA) plasma concentrations. The blood samples were centrifuged at 4°C within 15 minutes of collection, and the plasma was stored in liquid nitrogen until shipment on dry ice to Bangkok, where they were stored at −80°C until analysis. This entire procedure was performed for every patient for the first dose and in the subset of surviving and cooperative patients for the last dose of intravenous artesunate. The decision when to change from intravenous to oral antimalarial therapy was determined by the treating clinician.

The QTc interval was calculated using the mean QT interval, as measured manually over 10 complexes, corrected for heart rate using Bazett's,^11^ Fridericia's,^12^ the Framingham,^13^ Hodge's,^14^ and Price's^15^,^16^ corrections. Bazett's is the most frequently used but least satisfactory method, whereas Price's is the only method studied specifically in malaria and is thought to be the least sensitive to changes in heart rate.^3^ The JTc interval was calculated as QTc (Bazett's correction) minus QRS duration.

The primary outcome measure of the study was a change in QTc interval before and after artesunate. A significant change in QTc was defined as an increase in the interval in an individual patient after administration of artesunate to >500 ms^17^ or by an increase in the mean QTc of all patients by >25%.^4^ Secondary outcome measures were other significant changes in the ECG including JTc interval and arrhythmias, change in heart rate, and change in systolic blood pressure.

### Drug measurement.

Concentrations of artesunate and DHA in plasma were measured by the Clinical Pharmacology Laboratory in the Faculty of Tropical Medicine, Bangkok, by high-throughput liquid chromatographic tandem mass spectrometry (LC-MS/MS). Plasma samples were analyzed using solid-phase extraction (Oasis HLB, μ-elution plate; Waters, Milford, MA) and LC-MS/MS. Stable isotope-labeled artesunate and DHA were used as internal standards. Artesunate and DHA were quantified using an API 5000 triple quadripole mass spectrometer (Applied Biosystems/MDS SCIEX, Foster City, CA), with a TurboV ionization source (TIS) interface operated in the positive ion mode. Quantification was performed using selected reaction monitoring (SRM) for the transitions m/z 402–267 and 406–163 for artesunate and stable labeled artesunate, respectively, and 302–163 and 307–166 for DHA and stable labeled DHA, respectively. The liquid chromatographic (LC) system was an Agilent 1200 system (Agilent Technologies, Santa Clara, CA). Data acquisition and quantification were performed using Analyst 1.4 (Applied Biosystems/MDS SCIEX, Foster City, CA). The performance data for the assay during analysis of all samples expressed as coefficients of variation (CV%) for quality control samples were <5% throughout the calibration range.

### Pharmacokinetic analysis.

Pharmacokinetic analysis of the serial plasma artesunate and dihydroartemisinin concentrations was performed by non-compartmental analysis (NCA) using WinNonlin Version 5 (Pharsight, Mountain View, CA). Artesunate was assumed to be converted completely *in vivo* into DHA. NCA was performed using the linear/log trapezoidal method with extrapolation to time infinity for each individual patient using C_LAST_/λ_Z_, where C_LAST_ was the final concentration measurement, and λ_Z_ was the terminal elimination slope for each individual patient. The terminal elimination half-life was estimated by log linear regression of three to seven observed concentrations. Maximal artesunate and DHA plasma concentrations (C_max_) and time to maximal concentration (T_max_) were taken directly from the observed data.

### Statistical analysis.

Analysis was performed by Excel 2007 and STATA (version 9). Two-sided 95% confidence intervals (CIs) were calculated for mean QTc intervals and changes in QTc. Means were compared using a paired *t* test. Data were transformed to obtain a normal distribution if indicated, and correlation coefficients were calculated by the Pearson method. We calculated that a minimum of eight patients were needed to show an increase in mean QTc of 25% from baseline, with significance level (α) of 0.05 and a power (1 − β) of 0.90.

## Results

The study was conducted between late July 2006 and August 2007. In total, 21 adult patients were enrolled, of whom 11 had ECGs performed for both first and last doses of artesunate, and 10 were studied only after the first dose. The median number of doses of artesunate received intravenously was four, with a range of one to eight doses. Of the 21 patients, 8 (38%) died. The median (range) time to death was 36 (2–384) hours. Causes of death were deep coma caused by cerebral malaria (four patients), acute renal failure (two patients), aspiration pneumonia (one patient), and puerperal sepsis (one patient). There were no neurologic sequelae. Two surviving patients declined to have ECGs after the last dose of intravenous artesunate.

### Baseline characteristics and clinical outcome.

Baseline characteristics are summarized in Tables 1 and 2. Coma (GCS <11) was present in 13 patients (62%), and metabolic acidosis was common. Two patients (10%) were already in hemodynamic shock on admission, but only one patient required vasopressor drugs. Other complications during admission included acute renal failure (8/21), blackwater fever (2/21), and aspiration pneumonia (5/21). Three patients received a blood transfusion, and 10 patients were treated for suspected concomitant septicemia. Patients received a median of four doses (mean, 10.1 mg/kg for all patients and 11.6 mg/kg for survivors) of intravenous artesunate, followed by oral artemether-lumefantrine twice a day for a median of 3 days.

### QT interval.

**Figure 1.**
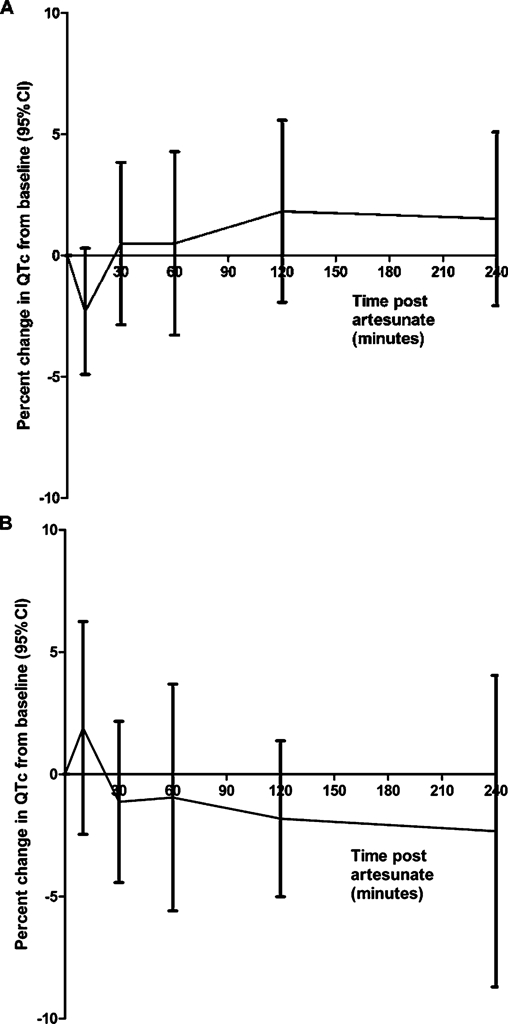
Percentage change in QTc (Bazett's correction with error bars showing upper and lower 95% confidence intervals) as a function of time after administration of the first (**A**) and last (**B**) doses of intravenous artesunate.

The mean QTc (Bazett) and change before and after administration of the first and last doses of intravenous artesunate are shown in Table 3. The percentage changes are shown in Figure 1. No significant change in the mean QTc was observed during the 240-minute observation period (*P* > 0.05), independent of the correction method used. Of the five QT correction methods used in this study, all showed similar patterns of change of QTc over time but with different magnitudes. The QTc from Bazett's correction was consistently the longest, followed in order by Hodges's, Price's, the Framingham, and Fridericia's. Although the Fredericia's method is less rate dependent, Bazett's correction was the most sensitive to detect changes in QTc in our dataset. The latter method was therefore chosen for use in Table 3 and the figures.

No patient had an increase of QTc from baseline of > 25%. The mean maximum increase in mean QTc (Bazett) after the first dose of artesunate was 5.8 ms (95% CI: 10.4–22.0 ms) at 120 minutes, and after the last dose, 7.8 ms (95% CI: 11.2–26.8 ms) at 10 minutes. There was no correlation between the total cumulative dose of artesunate or artemisinin received and the QTc (using all correction methods).

**Figure 2.**
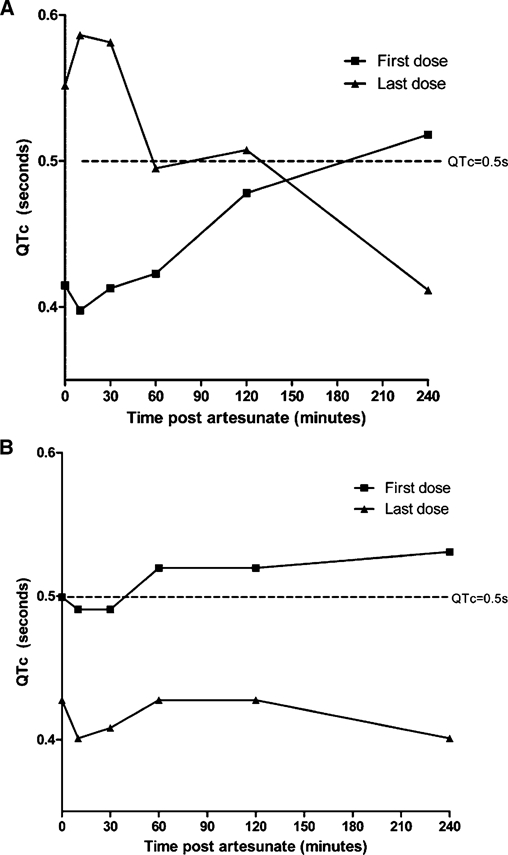
QTc as a function of time for patient X (**A**) and patient Y (**B**).

In two patients the QTc (Bazett) exceeded 0.5 seconds (Figure 2A for patient X and Figure 2B, for patient Y). Only Bazett's correction gave this result for the first doses for these patients, but all methods gave this result for the last dose for patient X.

Patient X, a 28-year-old man, had newly diagnosed peritoneal tuberculosis, but antituberculous chemotherapy had not started at the time of the study. The patient was cachectic with a body mass index of 12 kg/m^2^. On admission, the patient was in shock with a blood pressure of 70/40 mm of Hg and a temperature of 39.3°C. Blood pressure was restored to 90/70 mm of Hg after a fluid bolus, and the temperature decreased with paracetamol to 37.2°C at 4 hours after admission. The QTc (Bazett) was 0.415 seconds before the first dose of artesunate and did not increase significantly until 2 hours, rising to > 0.5 seconds only after 4 hours (0.518 seconds at 4 hours, an increase of 20%). The heart rate fell from 122 to 104 bpm over the same period. The serum potassium during this period remained stable at 5.1 mmol/L, with slightly low corrected serum calcium (8.1 mg/dL) and slightly high serum magnesium (mean, 31.2 mg/dL) throughout. In addition to artesunate, the patient also received one oral dose of 150 mg levamisole, 50 mg ranitidine intravenously, and from Days 3 to 5 after admission twice daily intravenous ceftriaxone (1 g) and metronidazole (500 mg) for suspected aspiration pneumonia. The last dose of intravenous artesunate was given on Day 6 after admission, at which time the patient had developed hypokalemia (2.3 mmol/L), which was corrected with 40 mmol of intravenous potassium over the first 4 hours plus oral potassium subsequently. Twenty-four hours later, the serum potassium had normalized at 4.2 mmol/L. The serum calcium and magnesium for this patient remained normal during this period. Before the last dose of intravenous artesunate on Day 6, the patient had a QTc (Bazett) of 0.552 seconds before artesunate administration, which rose to a maximum of 0.586 seconds 10 minutes after injection and fell to 0.411 seconds at 240 minutes after dose. The patient's heart rate rose from 82 to 115 bpm over these 4 hours. The prolonged QTc before and after the last dose of artesunate was most likely caused by hypokalemia.

Patient Y, a 26-year-old man, presented with nonoliguric acute renal failure (creatinine, 5.1 mg/dL) with metabolic acidosis (venous pH 7.301; base excess −13 mmol/L) and hypokalemia (2.9 mmol/L), which was initially not corrected. The QTc (Bazett) was borderline at 0.499 seconds before receiving the first dose of artesunate and increased 6% to 0.531 seconds by 240 minutes after administration of 2.4 mg/kg intravenous artesunate. During this period, this patient's corrected calcium remained normal (mean, 8.8 mg/dL), but serum magnesium fell slightly from 26.1 mg/dL at time 0 to 21.3 mg/dL at 4 hours, and heart rate fell from 110 bpm before artesunate injection to 100 bpm at 4 hours. It is possible this change in magnesium, together with hypokalemia, was the cause of the prolonged QTc in this patient. The QTc interval had normalized at Day 4 after admission, and serum potassium, calcium, and magnesium were normal at this time. Administration of artesunate had no effect on the QTc at this time. The patient had concomitant bronchopneumonia and cellulitis of the left leg and received intravenous ceftriaxone and flucloaxacillin from admission until Day 7. Peritoneal dialysis was started on Day 2.

At 0.33 (patient X) and 1.39 μg/mL (patient Y), the peak levels of artesunate measured in plasma from these two patients were below the upper quartile (1.88 μg/mL) of the range of values found in this study. This was also true for peak DHA levels except after the first dose in patient X (3.23 μg/mL; upper quartile, 2.77 μg/mL). Both patients survived.

### Other ECG changes.

There were no arrhythmias or changes in axis and no significant changes in JT interval, PR interval, or QRS duration after administration of either the first or last dose of artesunate. A possible extension of this study would be to use continuous ambulatory electrocardiographic recording in place of intermittent ECGs. However, in light of the negative findings from this study, it is unlikely that this will show additional and clinically significant cardiotoxicity.

### Heart rate and blood pressure.

There was no significant change in mean heart rate or blood pressure after administration of either the first or last dose of artesunate. Mean heart rate was significantly lower in patients receiving their last dose (89.0 bpm; 95% CI, 85.2–92.7 versus 113 bpm; 95% CI, 109–118; *P* < 0.0001), but mean systolic blood pressure was the same throughout (108 mm of Hg; 95% CI, 104–112 versus 111 mm of Hg; 95% CI, 108–115; *P* = 0.1). This change in heart rate was considered related to recovery, a decline in stress, and defervescence.^18^

### Artesunate/dihydroartemisinin pharmacokinetics.

The estimated pharmacokinetic parameters are shown in Table 4. The overall area under the drug concentration time curve (AUC; mean [min–max]) was 41.3 [4.37–216] and 121 µg/mL/min [32.1–240] for artesunate and dihydroartemisinin, respectively. The percentage of total AUC extrapolated to infinity was 3.37% [0.01–63] and 3.85% [0.07–31.9] for artesunate and dihydroartemisinin, respectively. The percentage of total AUC that was caused by back-extrapolation to estimate initial concentrations was 68.3% [10.6–96.8] for intravenous artesunate. Observed maximal artesunate and dihydroartemisinin plasma concentrations were 1.14 [0.14–4.40] and 2.11 µg/mL [0.59–3.76], respectively. Time to maximal concentration was 11.9 minutes [5.00–60.0] for dihydroartemisinin. There was no correlation between the number of doses of drug received and the differences in AUC and maximum concentration between the first and last dose.

**Figure 3.**
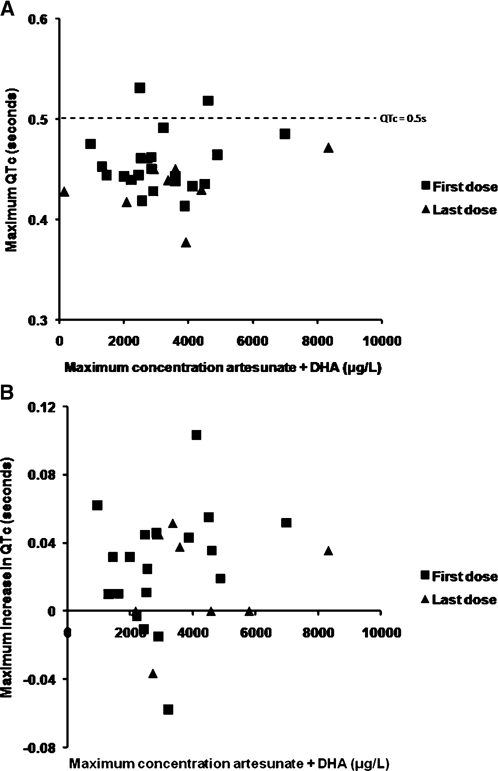
Maximum QTc (Bazett) (**A**) and maximum increase in QTc (Bazett) (**B**) as a scatter plot against maximum combined artesunate plus DHA concentration for each patient.

There were no significant correlations between the maximum QTc interval and the maximal plasma concentrations or AUC for artesunate, dihydroartemisinin, or the two combined. Scatter plots of maximum QTc and maximum increase in QTc against peak combined artesunate plus DHA concentration are shown in Figure 3A and B.

## Discussion

Although *Plasmodium falciparum* sequesters in the myocardial microvasculature, significant myocardial dysfunction or arrhythmias caused by the disease are very unusual in severe malaria. The quinoline antimalarials all have potent cardiovascular effects, but the effects of the artemisinin derivatives have been unclear. This study evaluated the effects of intravenous artesunate on the ECG in patients treated for severe malaria. Artesunate is given as a bolus injection, so very high blood levels occur at the end of the injection and the effects on cardiac conduction times can be assessed over a short time period, relatively independent of the changes in disease state. Even allowing for some hysteresis, resulting from equilibration from plasma to cardiac myocyte, these very high plasma concentrations would be expected to be associated with the greatest cardiovascular effects. However, there were no consistent cardiovascular or electrocardiographic effects after artesunate injection. In the acute phase of malaria, the QTc is commonly short because of increased sympathetic tone from arousal, stress, discomfort, anxiety, and usually fasting and elongates with recovery when the patient is relaxed, comfortable, supine in bed, and has often resumed eating. This relative QT prolongation has often been wrongly attributed to the effects of antimalarial drugs.^3^ Indeed, nearly all studies of antimalarial drugs (including sulfadoxine-pyrimethamine, which has no cardiac activity at all) report some prolongation of the QT interval in the days after the start of treatment.^3^ In this study, the QTc interval was unaffected by high doses of intravenous artesunate. There was no correlation between plasma levels of artesunate or its active metabolite dihydroartemisinin with QTc or the percentage increase in QTc. In addition, no effect was observed on the JTc or PR interval, QRS width, blood pressure, or heart rate.

There are many reasons underlying fluctuation in the QT interval in the acute phase of managing a life-threatening disease. In two patients, the QTc increased > 0.5 seconds. In one of these, the QTc was prolonged before the administration of artesunate and was related to hypokalemia. In the second patient, QTc did increase slightly after the administration of artesunate on admission. Because the QTc prolongation was not detected until 120 minutes after drug administration at a time that the artesunate and DHA concentrations had fallen to 0% and 18.7% of the respective peak values, and no change in QTc was detected at 10, 30, and 60 minutes, when DHA plus artesunate concentrations were 4.62, 2.82, and 1.71 µg/mL,it is unlikely that the QTc prolongation was caused by the artemisinins, even when assuming some degree of hysteresis in the system. Other drugs were also administered to this patient and together with defervescence during the observation period could have contributed to the prolongation in QTc time. It should be noted that administration of the same dose of artesunate to the same patient after recovery was associated with a decrease, rather than an increase, in QTc time, and in some other patients, QT decreased immediately after artesunate injection.

Of the antimalarial drugs, the quinolines, quinine, quinidine, chloroquine, and halofantrine, can cause significant prolongation of the QT interval. QT prolongation by 25% occurs in ~10% of patients given high-dose intravenous quinine.^10^,^19^ Halofantrine induces consistent dose-dependent prolongation of the QTc interval, and its use in patients with malaria has been associated with arrhythmias and sudden death.^4^ Although no longer recommended for the treatment of uncomplicated *falciparum* malaria in the absence of close cardiac monitoring, it is still available on the market. Most information on the cardiovascular effects, including ECG changes, of the artemisinin drugs are on the fat-soluble derivatives artemether and arteether, which have considerably longer plasma clearance times than water-soluble artesunate. In beagle dogs and rats, prolonged administration of high doses of artemether and arteether induced neurotoxicity, which was associated with a prolongation of the QT interval on the ECG.^9^,^20^,^21^ It is not clear, however, whether the effects on the ECG were a result of direct cardiotoxicity or were an indirect result of concomitant central nervous system toxicity. In a large randomized comparison of high-dose artemether and quinine in adults with severe malaria, serial ECGs were recorded in 301 patients. Slight QT prolongation was noted in both groups, 11 of 152 (7%) artemether recipients and 12 of 133 (9%) quinine recipients had an increase of > 25%.^10^ Neurotoxicity was not shown. There were no dysrhythmias or adverse cardiovascular effects in this study. The effects of recovery from severe malaria and any drug activity on ventricular repolarization could not be distinguished. Studies on oral administration of artemether, including in combination with lumefantrine, to both malaria patients and healthy volunteers did not show any significant electrocardiographic abnormalities including evidence of QTc prolongation.^18^,^22^–^25^ Compared with artemether and artemether, artesunate has a more favorable toxicity profile, even though plasma concentrations of the parent compound and the common metabolite DHA after intravenous injection are an order of magnitude higher. Considerably higher doses of artesunate are needed to produce neurotoxicity in animals.^9^,^26^,^27^ No effects on the ECG in dogs receiving the equivalent of the standard human dose of 2.4 mg/kg intravenous artesunate was observed.^28^ Doses several orders of magnitude higher than those used to treat malaria were required to cause negative inotropy in isolated guinea pig heart^29^ and hypotension in rabbits,^28^ all suggesting a very wide therapeutic ratio, and this is in line with the negative findings in this study.

In conclusion, this study showed that intravenous artesunate does not have significant cardiovascular effects in patients with severe *falciparum* malaria. High-dose intravenous artesunate does not prolong the QT interval.

## Figures and Tables

**Table 1:** Baseline characteristics of 21 patients with severe malaria

Variable	
Age, mean years (95% CI)	38.9 (32.9–44.9)
Number of male patients/number of female patients	17/4
Days of fever before admission, mean (95% CI)	6.12 (3.36–8.87)
Temperature, mean °C (95% CI)	37.6 (37.0–38.3)
Systolic blood pressure, mean mm of Hg (95% CI)	113 (99.1–127)
Heart rate, mean beats/min (95% CI)	111 (106–116)
Oxygen saturation, geometric mean % (95% CI)	94.2 (92.7–95.7)
Glasgow Coma Score, median (range)	10 (4–15)
Haemoglobin, mean g/dL (95% CI)	9.73 (8.60–10.9)
Peripheral WBC count, mean cells/mm^3^ (95% CI)	7,970 (5,100–10,900)
Platelet count, mean cells/mm^3^ (95% CI)	78,100 (33,400–123,000)
Parasitemia, geometric mean parasites/µL (95% CI)	125,000 (11,800–239,000)
Serum creatinine level, geometric mean mg/dL (95% CI)	1.99 (1.38–2.61)
Serum calcium level, mean mg/dL (95% CI)	8.38 (7.94–8.83)
Serum potassium level, mean mmol/L (95% CI)	4.23 (3.74–4.72)
Serum sodium level, mean mmol/L (95% CI)	136 (133–139)
Total serum bilirubin level, geometric mean µmol/L (95% CI)	3.77 (0.20–7.35)
Serum albumin, mean g/100 mL (95% CI)	3.09 (2.79–3.40)
Serum aspartate aminotransferase, geometric mean U/L (95% CI)	109 (73.7–144)
Serum alanine aminotransferase, geometric mean U/L (95% CI)	36.9 (23.1–50.7)
Serum glucose, geometric mean mg/dL (95% CI)	115 (68.3–147)
Serum base excess, mean mmol/L (95% CI)	−6.95) (−9.79 to −4.11)
Plasma lactate level, geometric mean mmol/L (95% CI)	3.94 (2.68–5.20)

**Table 2:** Distribution of presenting severity symptoms in patients with severe malaria

	Number of patients (%) (*N* = 21)	
Glasgow Come Scale <11	13	(61.9)
Haematocrit <20% with parasite count > 100,000/mm^3^	0	(0)
Bilirubin >3.0 mg/dL with parasite count > 100,000/mm^3^	2	(9.52)
Serum creatinine >3.0 mg/dL	5	(23.8)
Systolic blood pressure <80 mmHg with cool extremities	2	(9.52)
Peripheral asexual stage parasitemia >5%	3	(14.3)
Venous lactate >4 mmol/L	11	(52.4)
Venous bicarbonate <15 mmol/L	7	(33.3)

**Table 3:** Mean (95% CI) QTc (Bazett) and change from baseline after artesunate (*P* > 0.05)

Time post dose (min)	First dose		Last dose	
	QTc (ms)	Change in QTc from baseline (ms)	QTc (ms)	Change in QTc from baseline (ms)
0	424 (410–439)	0 NA	422 (391–453)	0 NA
10	414 (399–429)	−10 (−21 to 1)	430 (393–466)	8 (−11 to 27)
30	424 (409–439)	1 (−13 to 15)	424 (386–462)	−4 (−22 to 13)
60	424 (408–441)	1 (−15 to 17)	423 (400–446)	−6 (−27 to 15)
120	429 (416–443)	6 (−10 to 22)	416 (392–440)	−13 (−30 to 4)
240	429 (412–446)	5 (−10 to 21)	415 (399–431)	−14 (−46 to 18)

NA = not applicable.

**Table 4:** Pharmacokinetics of dihydroartemisinin (DHA) and artesunate shown as mean (95% CI) maximal observed plasma concentration (C_max_) and predicted area under the drug concentration time curve extrapolated to infinity (AUC)

Dose	DHA		Artesunate	
	C_max_ (μg/mL)	AUC (μg/mL/min)	C_max_ (μg/mL)	AUC (μg/mL/min)
First	2.06 (1.70–2.43)	121 (90.7–152)	1.02 (0.584–1.46)	45.1 (18.5–71.7)
Last	2.29 (1.93–2.64)	136 (94.4–178)	1.37 (0.390–2.34)	30.7 (13.4–48.1)
